# Cannabidiol and Cannabigerol Modify the Composition and Physicochemical Properties of Keratinocyte Membranes Exposed to UVA

**DOI:** 10.3390/ijms241512424

**Published:** 2023-08-04

**Authors:** Adam Wroński, Izabela Dobrzyńska, Szymon Sękowski, Wojciech Łuczaj, Ewa Olchowik-Grabarek, Elżbieta Skrzydlewska

**Affiliations:** 1 Dermatological Specialized Center “DERMAL” NZOZ in Białystok, Nowy Swiat 17/5, 15-453 Białystok, Poland; adam.wronski@dermal.pl; 2 Laboratory of Bioanalysis, Faculty of Chemistry, University in Białystok, Ciołkowskiego 1K, 15-245 Białystok, Poland; izadob@uwb.edu.pl; 3 Laboratory of Molecular Biophysics, Department of Microbiology and Biotechnology, Faculty of Biology, University in Białystok, Ciołkowskiego 1J, 15-245 Białystok, Poland; s.sekowski@uwb.edu.pl (S.S.); ewaolch@uwb.edu.pl (E.O.-G.); 4 Department of Analytical Chemistry, Medical University of Białystok, Mickiewicza 2D, 15-222 Białystok, Poland; elzbieta.skrzydlewska@umb.edu.pl

**Keywords:** phospholipids, ceramides, lipid rafts, membrane fluidity, membrane electrical charge, transmembrane transporters, phytocannabinoids

## Abstract

The action of UVA radiation (both that derived from solar radiation and that used in the treatment of skin diseases) modifies the function and composition of keratinocyte membranes. Therefore, this study aimed to assess the effects of phytocannabinoids (CBD and CBG), used singly and in combination, on the contents of phospholipids, ceramides, lipid rafts and sialic acid in keratinocyte membranes exposed to UVA radiation, together with their structure and functionality. The phytocannabinoids, especially in combination (CBD+CBG), partially prevented increased levels of phosphatidylinositols and sialic acid from occurring and sphingomyelinase activity after the UVA exposure of keratinocytes. This was accompanied by a reduction in the formation of lipid rafts and malondialdehyde, which correlated with the parameters responsible for the integrity and functionality of the keratinocyte membrane (membrane fluidity and permeability and the activity of transmembrane transporters), compared to UVA-irradiated cells. This suggests that the simultaneous use of two phytocannabinoids may have a protective effect on healthy cells, without significantly reducing the therapeutic effect of UV radiation used to treat skin diseases such as psoriasis.

## 1. Introduction

The skin acts as a barrier separating the interior of the human body from the environment, and normal skin functions are inextricably linked to the proper functioning of cellular membranes, especially those of cells that make up the epidermis. The phospholipids that make up the lipid bilayer play an important role in modulating the immune response and transducing signals to reach the interior of cells [1]. Within the lipid bilayer, in addition to phospholipids, there are specialized microdomains called lipid rafts, which are rich in cholesterol, sphingolipids and gangliosides, as well as proteins. These rafts are heterogeneous in both lipid and protein contents, while their structures are dynamic, resulting in continuous changes in the components that participate in physiological processes with different roles, including transport and receptor functions [2,3]. Therefore, it is believed that the disruption of physiological cellular functions results, among other factors, from changes in the lipid–protein structure of cell membranes and, thus, the number of functional groups on their surfaces. This causes changes in the electrical properties and fluidity of the skin cell membrane, which is also modified by exogenous factors [4], including UVA radiation, which accounts for up to 95% of all UV radiation and, as a part of sunlight, penetrates the atmosphere while traveling to the Earth’s surface [5]. UVA radiation reaches both the basal cells of the epidermis (keratinocytes) and cells of the dermis (fibroblasts) with blood vessels, also affecting dendritic, endothelial and immune cells [6]. Therefore, this radiation plays an important role in the proper functioning of the human body, including the initiation of the biosynthesis of biologically active compounds, as well as the induction of skin immunoprotection [7].

By increasing the generation of reactive oxygen species (ROS) and reducing the effectiveness of endogenous antioxidants [8], UVA radiation promotes a pro-inflammatory environment in keratinocytes [9]. The consequences include oxidative modifications of cellular components, including lipids and cellular proteins [8,10,11]. Among the preferential targets of ROS action are membrane phospholipids, leading to their peroxidation and, by increasing the activity of lipolytic enzymes, an increase in their enzymatic metabolism. This leads to increased levels of lipid mediators, including endocannabinoids and eicosanoids, which play important roles in redox and inflammatory processes [12,13]. Moreover, in relation to cell membranes, this disrupts the lipid bilayer structure, which in turn may contribute to modifications of membrane properties, such as electrical charge and membrane fluidity [14]. Altered membrane fluidity can also affect the functions of membrane proteins by modifying their lipid microenvironments and their interactions with other membrane components, thereby altering membrane functionality [15,16]. To prevent such consequences, there is a constant search for compounds, especially lipophilic compounds of natural origin, that easily penetrate the lipid bilayer and are characterized by the ability to prevent metabolic disorders under UVA radiation.

The pharmacotherapeutic potential of secondary metabolites of *Cannabis sativa* L., such as phytocannabinoids, has been of great research interest in the last decade due to their chemical properties and associated biological effects. One of the best-studied phytocannabinoids in the context of pharmacotherapy is cannabidiol (CBD), which can modulate intracellular redox and inflammatory signaling [17,18], both through the direct regulation of ROS production and by regulating the expression of the transcription factors Nrf2 and NFκB. In addition, CBD, by influencing the activity of enzymes that metabolize phospholipids to endocannabinoids, which activate membrane receptors coupled with G proteins, especially CB1 and CB2, indirectly participates in the regulation of ROS and TNFα levels [17]. Even more effective appears to be another phytocannabinoid, cannabigerol (CBG), which effectively reduces UVA/B-radiation-elevated ROS levels [19]. Interestingly, other metabolic actions of this compound have not yet been as thoroughly studied as those of CBD. However, CBG has been found to inhibit the release of pro-inflammatory cytokines in UV-treated cells more potently than CBD [19]. Consequently, the antioxidant effect of phytocannabinoids promotes a reduction in oxidative stress and its UV-induced consequences and prevents inflammation [20], ultimately providing photoprotection to skin cells. This is even more important, as UV radiation is used therapeutically in the treatment of some skin diseases, including psoriasis [21].

The present study aimed to evaluate the effects of CBD and CBG, both alone and in combination, on the lipid components of the cell membranes of keratinocytes exposed to UVA irradiation. The use of these compounds may consequently contribute to the protection of the intracellular metabolism of these cells.

## 2. Results

**Figure 1:**
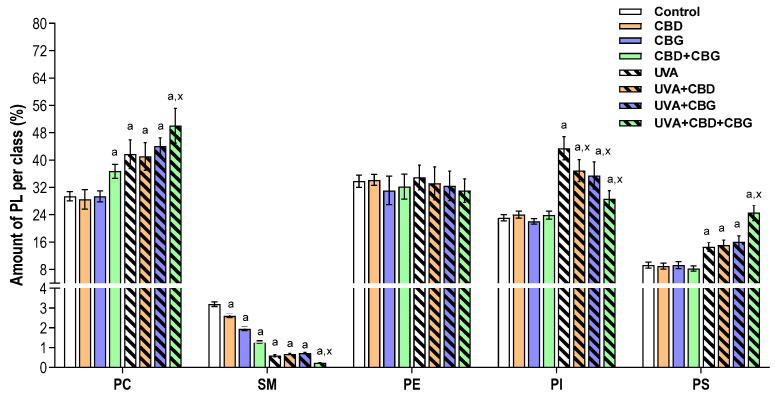
Relative phospholipid classes in the following keratinocyte groups: control; cultured with CBD (5 μM) for 24 h; cultured with CBG (1 μM) for 24 h; cultured with CBD (5 μM) and CBG (1 μM) for 24 h; irradiated with UVA (30 J/cm^2^); irradiated with UVA (30 J/cm^2^) and cultured with CBD (5 μM) for 24 h after irradiation; irradiated with UVA (30 J/cm^2^) and cultured with CBG (1 μM) for 24 h; irradiated with UVA (30 J/cm^2^) and cultured with CBD (5 μM) and CBG (1 μM) for 24 h. Values are mean ± SD, *p* < 0.05; a, significantly different from control; x, significantly different from UVA group.

Univariate analysis (one-way ANOVA and Tukey’s post hoc test) was used to assess the variation in the relative abundance of the phospholipid classes under the study conditions (Figure 1). We found that PCs were upregulated in non-irradiated keratinocytes treated with both CBD and CBG. Moreover, a general tendency toward a decrease in relative SM content was observed after CBD or CBG treatment. However, when compared with control cells, a significant downregulation of SM was observed in keratinocytes treated with both CBD and CBG (Figure 1).

Significant changes in the phospholipid profile were also observed in keratinocytes irradiated with UVA when compared to control cells. These changes included the upregulation of PC and the downregulation of SM. In addition, keratinocytes exposed to UVA radiation demonstrated an upregulation of both PS and PI. No significant differences were found between the phospholipid profiles of UVA-irradiated keratinocytes after CBD or CBG treatment, with the exception of the downregulation of PI (Figure 1). However, it should be noted that in comparison with non-irradiated keratinocytes, the exposure of these cells to both phytocannabinoids simultaneously (CBD+CBG) led to the dramatic downregulation of both PI and SM. In addition to these changes, we also found a significant upregulation of PC in UVA-irradiated cells following treatment with both CBD and CBG when compared with UVA-irradiated cells (Figure 1).

**Figure 2:**
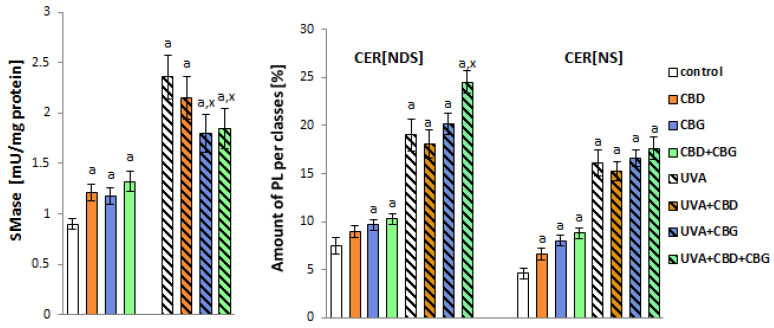
SMase activity panel and relative ceramide content within CER[NDS] and CER[NS] classes in the following keratinocyte groups: control; cultured with CBD (5 μM) for 24 h; cultured with CBG (1 μM) for 24 h; cultured with CBD (5 μM) and CBG (1 μM) for 24 h; irradiated with UVA (30 J/cm^2^); irradiated with UVA (30 J/cm^2^) and cultured with CBD (5 μM) for 24 h after irradiation; irradiated with UVA (30 J/cm^2^) and cultured with CBG (1 μM) for 24 h; irradiated with UVA (30 J/cm^2^) and cultured with CBD (5 μM) and CBG (1 μM) for 24 h. Values are mean ± SD, *p* < 0.05; a, significantly different from control; x, significantly different from UVA group.

Considering the results obtained in ceramide profiling, we focused on ceramides containing non-hydroxy fatty acids and sphingosine (CER[NS]) and ceramides containing non-hydroxy fatty acids and dihydrosphingosine (CER[NDS]). These were the most abundant and represented the two main CER classes among all ceramide species identified in all examined keratinocyte groups (Figure 2).

Our results demonstrated a significant upregulation of CER[NDS] and CER[NS] in keratinocytes treated with CBG and with both CBG and CBD, accompanied by an increase in the activity of SMase observed in these experimental groups compared with the control group. Interestingly, the treatment of control keratinocytes with CBD led to a significant increase in the level of CER[NS] but not CER[NDS]. The exposure of keratinocytes to UVA caused a dramatic increase in both the activity of SMase and the contents of ceramides belonging to both classes. However, we observed an additional upregulation of CER[NDS] in UVA-irradiated keratinocytes after treatment with both CBD and CBG (Figure 2).

**Figure 3:**
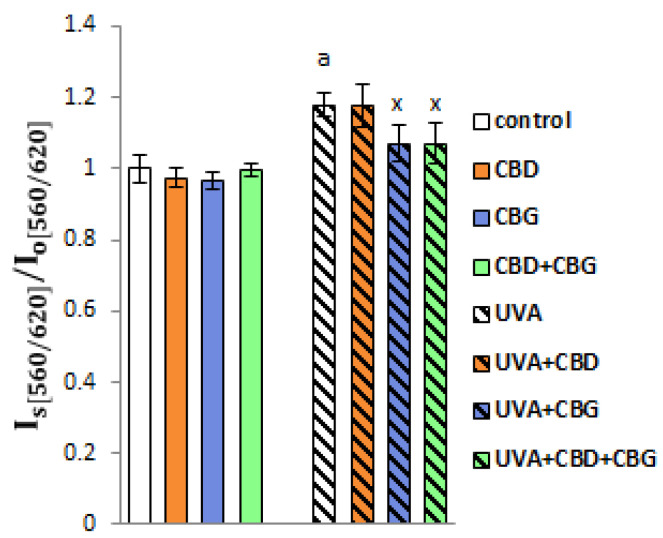
Formation of lipid rafts in the following keratinocyte groups: control; cultured with CBD (5 μM) for 24 h; cultured with CBG (1 μM) for 24 h; cultured with CBD (5 μM) and CBG (1 μM) for 24 h; irradiated with UVA (30 J/cm^2^); irradiated with UVA (30 J/cm^2^) and cultured with CBD (5 μM) for 24 h after irradiation; irradiated with UVA (30 J/cm^2^) and cultured with CBG (1 μM) for 24 h; irradiated with UVA (30 J/cm^2^) and cultured with CBD (5 μM) and CBG (1 μM) for 24 h. Values are mean ± SD, *p* < 0.05; a, significantly different from control; x, significantly different from UVA group. I_s_ is the ratio λem.1/λem.2 for samples, while I_0_ is the ratio λem.1/λem.2 for the control cells.

A similar change was observed in the tendency of lipid rafts to form in keratinocytes. Phytocannabinoids applied either alone or together did not affect lipid raft formation in the control keratinocytes (Figure 3). Conversely, a statistically significant increase in the lipid order parameter was found after UVA irradiation, indicating enhanced lipid raft formation. The introduction of either CBG alone or CBD+CBG in keratinocytes after UVA irradiation reduced the tendency for lipid raft formation. In contrast, the application of CBD alone did not affect lipid raft formation in either the control or UVA-exposed keratinocytes.

**Figure 4:**
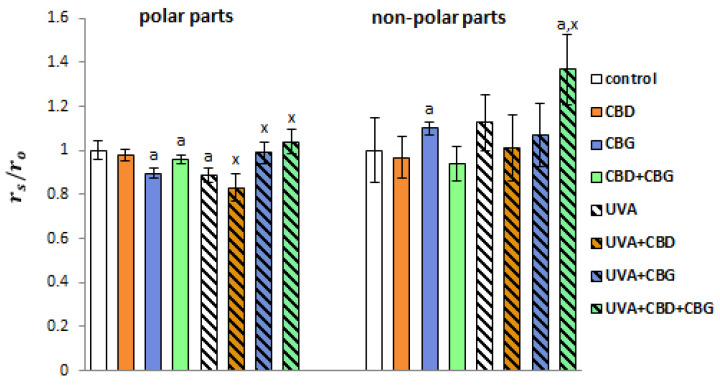
Fluorescence anisotropy of membrane phospholipids in their polar (TMA-DPH staining) and non-polar (DPH staining) parts in the following keratinocyte groups: control; cultured with CBD (5 μM) for 24 h; cultured with CBG (1 μM) for 24 h; cultured with CBD (5 μM) and CBG (1 μM) for 24 h; irradiated with UVA (30 J/cm^2^); irradiated with UVA (30 J/cm^2^) and cultured with CBD (5 μM) for 24 h after irradiation; irradiated with UVA (30 J/cm^2^) and cultured with CBG (1 μM) for 24 h; irradiated with UVA (30 J/cm^2^) and cultured with CBD (5 μM) and CBG (1 μM) for 24 h. Values are mean ± SD, *p* < 0.05; a, significantly different from control; x, significantly different from UVA group. The results are presented as the r_s_/r_0_ ratio, where r_s_ and r_0_ are the anisotropy values for the test and control samples, respectively.

Fluorescence measurements using cell staining with TMA-DPH (anchored to the polar parts of the membrane) and DPH (attached deeper in the hydrophobic, non-polar parts of phospholipids) were used to study the fluidity of keratinocyte cell membranes (Figure 4). It was shown that UVA irradiation caused a significant decrease in anisotropy in the polar regions of the keratinocyte membrane, indicating increased fluidity. The introduction of CBD into the medium after UVA irradiation resulted in a greater decrease in membrane rigidity (i.e., an increase in fluidity), while the opposite effect was observed after CBG and CBD+CBG administration compared with cells that were UVA-irradiated only.

**Figure 5:**
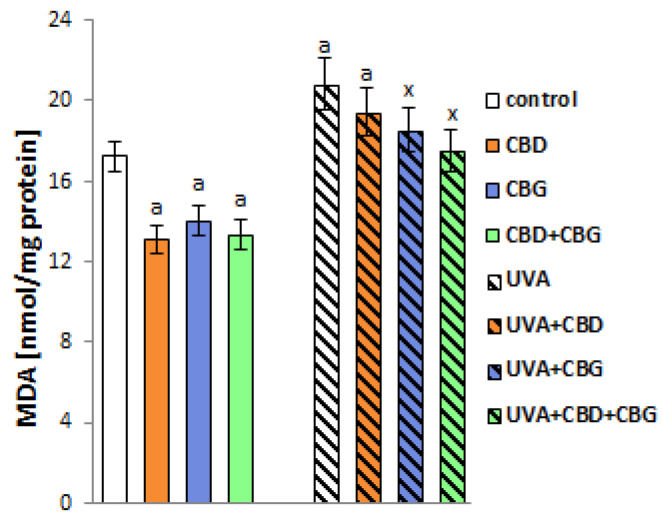
The levels of lipid peroxidation product (MDA) in the following keratinocyte groups: control; cultured with CBD (5 μM) for 24 h; cultured with CBG (1 μM) for 24 h; cultured with CBD (5 μM) and CBG (1 μM) for 24 h; irradiated with UVA (30 J/cm^2^); irradiated with UVA (30 J/cm^2^) and cultured with CBD (5 μM) for 24 h after irradiation; irradiated with UVA (30 J/cm^2^) and cultured with CBG (1 μM) for 24 h; irradiated with UVA (30 J/cm^2^) and cultured with CBD (5 μM) and CBG (1 μM) for 24 h. Values are mean ± SD, *p* < 0.05; a, significantly different from control; x, significantly different from UVA group.

These results confirmed previous reports [4] showing that the UVA irradiation of keratinocytes is accompanied by a shift in the redox balance toward oxidative conditions, increasing the level of the classic lipid peroxidation product MDA (Figure 5) by approximately 20% compared with controls. The application of CBD, CBG and CBD+CBG together reduced MDA levels in unexposed keratinocytes by approximately 30%, 25% and 30%, respectively. However, following UVA irradiation, the phytocannabinoids reduced MDA levels by approximately 15% and 20% compared to irradiated cells for CBG and CBD+CBG, respectively. Interestingly, there were no significant changes observed after CBD administration.

**Figure 6:**
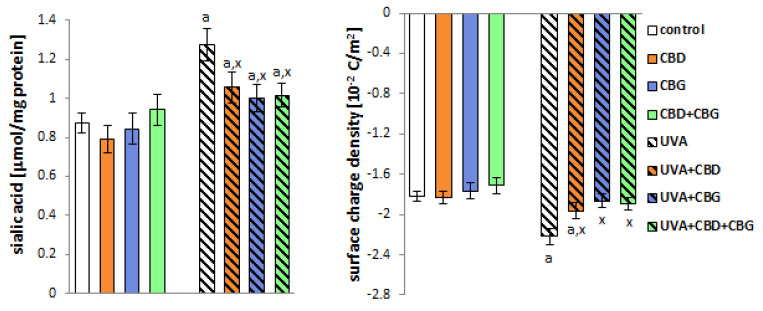
The sialic acid content and the surface charge density of the following keratinocyte groups: control; cultured with CBD (5 μM) for 24 h; cultured with CBG (1 μM) for 24 h; cultured with CBD (5 μM) and CBG (1 μM) for 24 h; irradiated with UVA (30 J/cm^2^); irradiated with UVA (30 J/cm^2^) and cultured with CBD (5 μM) for 24 h after irradiation; irradiated with UVA (30 J/cm^2^) and cultured with CBG (1 μM) for 24 h; irradiated with UVA (30 J/cm^2^) and cultured with CBD (5 μM) and CBG (1 μM) for 24 h. Values are mean ± SD, *p* < 0.05; a, significantly different from control; x, significantly different from UVA group.

The introduction of CBD and/or CBG into control keratinocytes’ media did not affect the levels of sialic acid, which is one of the main components of cell membrane glycolipids and glycoproteins. In contrast, and in addition to altering the composition of membrane phospholipids, UVA radiation increased sialic acid levels by approximately 45% compared to control cells (Figure 6). However, the application of CBD, CBG and CBD+CBG after UVA irradiation significantly reduced sialic acid levels compared with UVA-exposed cells by approximately 20%, 25% and 25%, respectively. The direction of the change in the level of sialic acid corresponds to the direction of the change in the electric charge of the keratinocyte membrane (Figure 6). The negative electrical charge of the membrane increased after the UVA irradiation of cells by approximately 20% compared to the control cells. By contrast, the introduction of CBD and CBG individually and in combination into the cell medium after UVA irradiation of cells reduced the negative charge of the membrane by approximately 15%, 20% and 20%, respectively, compared with cells irradiated with UVA alone.

**Figure 7:**
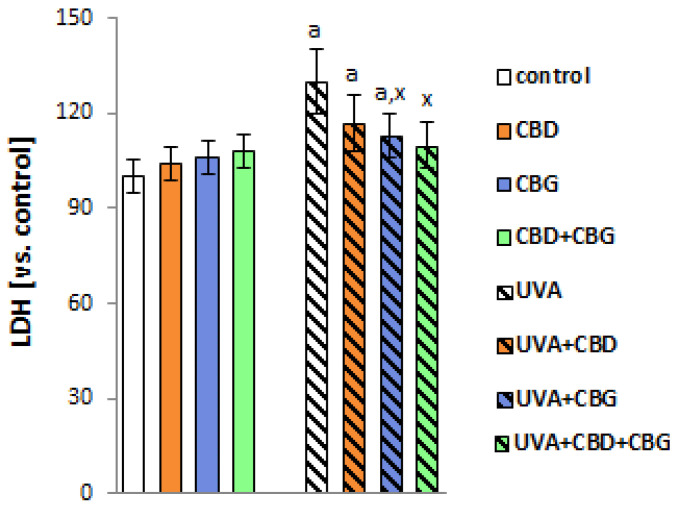
The activity of lactate dehydrogenase (LDH) in the media of the following keratinocyte groups: control; cultured with CBD (5 μM) for 24 h; cultured with CBG (1 μM) for 24 h; cultured with CBD (5 μM) and CBG (1 μM) for 24 h; irradiated with UVA (30 J/cm^2^); irradiated with UVA (30 J/cm^2^) and cultured with CBD (5 μM) for 24 h after irradiation; irradiated with UVA (30 J/cm^2^) and cultured with CBG (1 μM) for 24 h; irradiated with UVA (30 J/cm^2^) and cultured with CBD (5 μM) and CBG (1 μM) for 24 h. Values are mean ± SD, *p* < 0.05; a, significantly different from control; x, significantly different from UVA group.

Changes in the composition of keratinocyte cell membranes following exposure to UVA affected cell function. We observed that after UVA, the membrane permeability of keratinocytes increased by approximately 30%, based on LDH leakage from the cells (Figure 7). The introduction of CBG into keratinocyte media individually and in a CBD+CBG system after UVA irradiation reduced this leakage by approximately 15% and 20%, respectively. It can therefore be concluded that CBG administered both alone and together with CBD reduces the degree of membrane damage to keratinocytes following radiation exposure.

**Figure 8:**
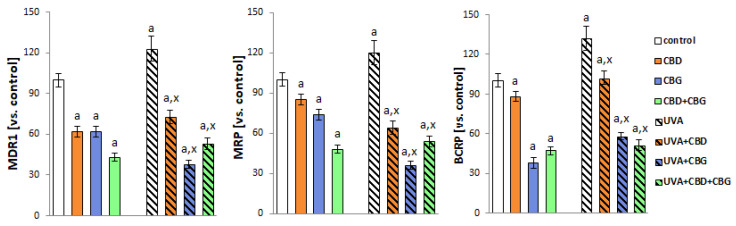
The activity of ABC-cassette transporters (MDR1, MRP, BCRP) in the following keratinocyte groups: control; cultured with CBD (5 μM) for 24 h; cultured with CBG (1 μM) for 24 h; cultured with CBD (5 μM) and CBG (1 μM) for 24 h; irradiated with UVA (30 J/cm^2^); irradiated with UVA (30 J/cm^2^) and cultured with CBD (5 μM) for 24 h after irradiation; irradiated with UVA (30 J/cm^2^) and cultured with CBG (1 μM) for 24 h; irradiated with UVA (30 J/cm^2^) and cultured with CBD (5 μM) and CBG (1 μM) for 24 h. Values are mean ± SD, *p* < 0.05; a, significantly different from control; x, significantly different from UVA group.

After UVA exposure, the activity of multiple ABC-cassette transporters (MDR1, MRP, BCRP) increased significantly (Figure 8). However, the introduction of CBD and CBG alone and in combination into keratinocytes after UVA irradiation decreased the activity of MDR1, MRP and BCRP compared with UVA-irradiated cells.

**Figure 9:**
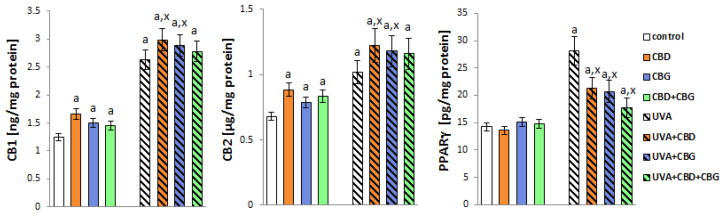
The expression of receptors (CB1, CB2, PPARγ) in the following keratinocyte groups: control; cultured with CBD (5 μM) for 24 h; cultured with CBG (1 μM) for 24 h; cultured with CBD (5 μM) and CBG (1 μM) for 24 h; irradiated with UVA (30 J/cm^2^ ); irradiated with UVA (30 J/cm^2^) and cultured with CBD (5 μM) for 24 h after irradiation; irradiated with UVA (30 J/cm^2^) and cultured with CBG (1 μM) for 24 h; irradiated with UVA (30 J/cm^2^) and cultured with CBD (5 μM) and CBG (1 μM) for 24 h. Values are mean ± SD, *p* < 0.05; a, significantly different from control; x, significantly different from UVA group.

The decrease in the activity of keratinocyte membrane transporters under the influence of the phytocannabinoids used was accompanied by an increase in the expression of G-protein-coupled receptors (CB1/2 and PPARγ) (Figure 9). The exposure of cells to UVA radiation significantly increased the expression of the above-mentioned receptors. However, the phytocannabinoids used, especially in the CBD+CBG system, did not affect the expression of CB1 and CB2, but they significantly reduced the expression of the PRAPγ receptor in UVA-treated keratinocytes.

## 3. Discussion

The integrity of cell membranes, resulting from their physiological composition, ensures both proper intracellular metabolism and intercellular communication [22]. The main structural element of the cell membrane is the phospholipid bilayer, containing sphingolipid- and cholesterol-enriched lipid rafts, which determine its structure and facilitate signal transduction and intracellular transport [2]. Keratinocytes, which are the major cell type in the epidermis, are constantly exposed to physicochemical environmental factors, including solar radiation containing UVA. This environmental insult significantly affects the metabolism of keratinocytes, including the redox balance and inflammatory processes [23]. The resulting pro-oxidative conditions promote modifications to the components of keratinocyte membranes, including lipids and proteins, resulting in changes to both the structure and function of cell membranes and impaired intracellular metabolism [24].

Our previous results [25,26] and the present study show that UVA radiation promotes the upregulation of keratinocyte membrane phospholipids, such as PC, PS and PI, and the downregulation of SM, with no changes in PE content. The reduction in SM content under the influence of UVA is related to the increased metabolism of SM because of increased SMase activity. These observations are consistent with the literature indicating that UVA radiation increases the mRNA expression of acidic and neutral SMases [27]. Given the increase in ceramide levels, the stimulation of sphingomyelin–ceramide pathways may be suggested, since the enzymatic hydrolysis of sphingomyelin is one of the main mechanisms leading to the formation of ceramides and phosphocholines [28]. In addition, the phosphocholine formed in these transformations can be further used for the synthesis of PC [28], which may be the reason for the increase in the relative content of PC observed following UVA irradiation in this study. Similar effects were also observed when SM levels were decreased by inhibitors of sphingolipid synthesis or SMase action [29]. The UV irradiation of keratinocytes also promotes the generation of pro-inflammatory cytokines (interferon-γ, TNF-α and IL-1ß), which may lead to increased ceramide synthesis resulting from SMase activation [30,31]. Moreover, previous data confirm that an increase in ceramide levels is observed as a consequence of the increased activity of acid SMase, which leads to the increased hydrolysis of SM in human skin cells [32]. In turn, ceramides provide a platform for the accumulation and activation of cytokine receptors, including TNFR1 receptors and ion channels, specifically the potassium channel Kv1.3 and the calcium-release-activated calcium channel (CRAC) [33,34].

The changes observed in the composition of the lipid bilayer may be the reason for the reorganization of the cell membrane structure, including the increased movement of PS to the outer layer of the cell membrane. This was indicated by the change in the surface membrane charge and increased cholesterol efflux from the cells, both dependent on and independent of the ABCA1 transporter [29]. Regardless of the qualitative and quantitative changes in the structure of phospholipids, there is an increase in the total level of sialic acid following UVA exposure. Sialic acid is a component of glycolipids and glycoproteins and carries a negative charge on the surface of keratinocyte membranes. Previous data support a link between increased sialic acid levels and the UVA-induced enhancement of membrane sialylation [35], which plays an important role in cell signaling [36], cell adhesion [37] and cell–cell communication [38]. Changes in surface glycosylation, especially in terminal sialic acids, can also directly enhance phagocytosis to regulate apoptotic cell clearance [38]. In addition, sialic acid can bind to Siglec receptors on the surface of lymphocytes and inhibit immunoglobulin receptor (BCR)-mediated signaling [39]. A consequence of changes in both phospholipid composition and sialic acid levels in keratinocyte membranes is a different number of functional groups on the membrane surface and, consequently, changes in the electrical properties of the membrane, which is confirmed by the present and previous results [4,40]. UVA radiation causes an increase in the negative charge on the surface of UVA-exposed keratinocytes compared with controls, which corresponds to an increase in sialic acid content and the higher exposure of phosphatidylserine on the outer surface of the cell membrane [41]. Changes in the quantity and type of polar groups on the cell membrane surface and the increased number of lipid rafts [42] contribute to the increased fluidity of the polar part of the membrane, a finding confirmed by our results. Conversely, the previous literature and our observations herein indicate that the increased levels of ceramides and cholesterol lead to an increase in the formation of sphingolipid- and cholesterol-rich microdomains (lipid rafts) in cell membranes, which promote their integrity [43]. However, this situation can exacerbate inflammatory processes and immunosuppression [2]. The increase in lipid raft formation under UVA radiation can also be associated with a mechanism that triggers cell apoptosis, as UV radiation is one of the stimuli that modify the components of lipid rafts (like the above-mentioned PC and SM) but also affect gene expression and control apoptosis via the aggregation of the Fas protein and the promotion of the Death-Inducing Signaling Complex (DISC) [44]. However, other inflammatory mechanisms cannot be ruled out, as it is well known that UV induces many pro-inflammatory factors, including COX-2, TNF-α, IL-6, IL-10, IL-12, iNOS and others [24]. This can lead to the formation of so-called inflammarafts, which are enlarged lipid rafts [45], as a reaction to inflammatory factors induced by UV radiation. Although the concept of inflammarafts has been described for glial cells [45], it cannot be ruled out that they can also be formed in the keratinocyte membrane, especially given that their development was also observed by Navia-Pelaez in macrophage cells [46]. The increased generation of ROS accompanying the exposure of keratinocytes to UVA radiation [23] may also cause an increase in the number of lipid rafts, which was observed, for example, in T lymphocytes [47]. At the same time, however, UVA radiation promotes enhanced ROS reactions, both with phospholipid PUFAs, with the formation of cyclic prostaglandin derivatives, and with free PUFAs, derived from the reaction of lipolytic enzymes with phospholipids to form low-molecular peroxidation products, namely, aldehydes, including MDA [48,49], which is confirmed by our current results. Therefore, the structure and function of cell membranes are modified, including the disruption of membrane permeability. In turn, the resulting highly reactive protein-binding unsaturated aldehydes disrupt cellular antioxidant capacity and intracellular signaling, affecting intracellular metabolism [50,51].

On the other hand, changes in keratinocyte membrane permeability are also associated with the increased expression of ABC membrane transporters, including BCRP, under the influence of UVA radiation. The literature data indicate that the oxidative stress that accompanies the UVA irradiation of keratinocytes [13] promotes the activation of these ABC transporters. This facilitates the transport of both exogenous and endogenous substances across membranes, which may additionally disturb cellular metabolism [52]. However, it is known that BCRP, in addition to its protective role against toxins, significantly stimulates the differentiation of immune cell activation in skin cells in response to harmful environmental factors [53].

To prevent the effects of the modified composition and organization of the keratinocyte membrane structure under the influence of UV radiation, both as a component of sunlight and as the irradiating agent used in the phototherapy of skin diseases such as psoriasis, effective therapeutic solutions are sought, especially those based on the use of natural compounds, such as phytocannabinoids [4,54]. Being predominantly lipophilic, they easily penetrate the lipid bilayer to protect the components of cell membranes; in addition, by penetrating the cytosol, they also protect against metabolic disorders [13]. Regardless of the possibility of the penetration of lipophilic phytocannabinoids through the phospholipid structures of keratinocyte membranes, they can be transported, like other compounds, by ABC transporters that participate in the supply–removal of both exogenous and endogenous compounds. The results of this study indicate that the used phytocannabinoids reduce the transport across the membranes of keratinocytes, both control and, above all, UVA-irradiated. This reduces the absorption and removal of potentially toxic substances [13]. Therefore, it is natural for the expression of transporters to increase after UVA irradiation and to decrease after the use of phytocannabinoids. The regulation of protein expression and transport depends on several factors, including the Nrf2 transcription factor, the effectiveness of which depends, among others, on activators and inhibitors, as well as on NFkB [55], the level of which is modified by both UVA radiation and the phytocannabinoids used [56]. Therefore, the presented results also indicate the regulatory role of phytocannabinoids, especially those acting as a team, in regulating membrane transport. BCRP deficiency has also been shown to reduce the PPIX distribution in the skin and thus prevent EPP-related phototoxicity [57].

Based on the previous literature, it can be suggested that the presence of hydroxyl groups at positions 1 and 3 of the phenolic ring in the structures of CBD and CBG provides the ability to interrupt free radical chain reactions by trapping free radicals or converting radicals into less active forms [58,59]. It is also known that CBD, by chelating the transition metal ions involved in the Fenton reaction, also reduces the UVA-enhanced generation of extremely reactive hydroxyl radicals [60]. In addition to the direct reduction in ROS levels and the stabilization of the redox balance, both CBD and CBG also act indirectly through the modulation of endocannabinoid metabolism (anandamide, AEA, and 2-arachidonoylglycerol, 2-AG) and the activation of G-protein-coupled and peroxisome proliferator-activated membrane receptors, which may play a key role in the regulation of both the redox balance and inflammation [58,61,62,63]. CBD and CBG are known to have a weak affinity for the cannabinoid receptor CB1, whose activation results in the increased production of ROS and TNFα and promotes oxidative stress and inflammation [64]. In contrast, as CBG is a strong agonist and CBD is a weak CB2 antagonist, these two phytocannabinoids have different abilities to activate these receptors [59,65]. Consequently, CBG promotes a reduction in ROS and TNFα generation, thereby enhancing the antioxidant and anti-inflammatory responses [66]. This may be the reason for the varied responses of membrane components to the application of CBD, CBG and CBD+CBG. Since phospholipids, belonging to the PC class, are the main reservoir of unsaturated fatty acids susceptible to oxidation, the increase in PC content observed in UVA-irradiated keratinocytes treated with CBD and CBG may be a result of the antioxidant properties of these compounds and decreased lipid peroxidation in these cells. The treatment of UVA-irradiated keratinocytes with phytocannabinoids (CBD and CBG, separately and together) partially prevented the upregulation of PI resulting from irradiation. Interestingly, the greatest PI reduction was observed after the exposure of cells to both phytocannabinoids simultaneously, and similar changes were observed in the content of sialic acid. Reducing the amount of this acid caused changes in the number of negatively charged groups on the membrane surface and thus reduced the negative charge of the keratinocyte membrane and membrane fluidity, especially in the polar portion. Both the results of this work and the previous literature [67] show that CBG has slightly (but often statistically significantly) better protective properties than CBD. It is believed that CBG’s ability to regulate lipid metabolism is primarily related to its effect on PPAR receptors. CBG has been shown to have a greater affinity for the PPARγ receptor than CBD [68,69], which would explain the intense changes in the phospholipid profile of keratinocytes treated with this compound. In addition, the action of phytocannabinoids on cannabinoid receptors inhibits adenylyl cyclase and voltage-gated calcium channels, as well as kinases and potassium channels, including mitogen-activated protein kinase (MAPK) and phosphatidylinositol 3-phosphatidyl kinase (PI3K)/AKT [70]. It should be stressed that the PI3K/AKT/mTOR pathway is one of those required for protein synthesis and the induction of other intercellular pathways, such as the MAPK pathway, which plays an important role in the regulation of cell survival, proliferation and apoptosis [71]. The phytocannabinoids used in the present study exhibit antioxidant and anti-inflammatory effects [58,66,72], which can be used in the treatment of skin diseases such as psoriasis [55,62]. The results of this study confirm that the application of CBD and CBG separately and in combination reduces the severity of oxidative stress, which was assessed based on the level of MDA, which was decreased in both unexposed and UVA-irradiated keratinocytes.

Other studies [73] have shown that CBD incorporated into cell membranes changes the orientation of cholesterol in the phospholipid environment and reduces its transverse diffusion. This, in turn, alters the biophysical properties of cell membranes, including their fluidity, as shown in this study, and affects the function of proteins, especially transmembrane proteins, which are regulated by lipid rafts and whose formation in UVA-irradiated keratinocytes is inhibited specifically by the use of CBG and CBG+CBD. This may suggest the higher effectiveness of CBG, especially in a lipophilic environment. This effect is also confirmed by changes in the levels of the phospholipid classes and peroxidation product (MDA) observed in our studies. This is also confirmed by more intense changes in the activity of membrane transporters (ABC). This assumption can be considered consistent with literature data, including, e.g., a study showing that the release of pro-inflammatory cytokines induced by UVA radiation in human dermal fibroblasts (HDFs) and normal human epidermal keratinocytes (NHEKs) was inhibited more strongly by CBG than by CBD [19]. Also, CBG, compared to CBD, showed a stronger antibacterial effect [74]. The effect of phytocannabinoids on proteins may relate to both transcriptional and post-transcriptional interactions, which may partly explain the higher expression of collagen I, elastin and fibronectin found after the use of CBD [19]. In addition, the enhanced effects of both tested phytocannabinoids can be attributed to the synergistic or additive effects of these two compounds, as confirmed by changes in the levels of phospholipid classes and their peroxidation product. This is confirmed by the results of other authors indicating that CBD promotes the anti-inflammatory effect of CBG [75], and the interaction between both phytocannabinoids reduces the viability of glioblastoma cells [76].

## 4. Materials and Methods

### 4.1. Cell Culturing and Treatment

Studies were performed on immortalized human keratinocytes (CDD 1102 KERTr), which were purchased from the American Type Culture Collection ATCC^®^ (Manassas, VA, USA). Keratinocytes from passage 10 were used for the study. Keratinocytes were cultured in Keratinocyte Serum-Free Medium (Gibco, Grand Island, NY, USA) containing epidermal growth factor (EGF 1-53) (5 µg/L), fetal bovine serum (10%) and antibiotics (50 U/mL penicillin and 50 μg/mL streptomycin). All cell culture experiments were performed under sterile conditions, including sterile plastics and cell culture reagents purchased from Gibco (Grand Island, NY, USA). After reaching 70% confluence, the keratinocytes were exposed to UVA radiation (365 nm, 30 J/cm^2^) (Bio-Link Crosslinker BLX 365, Vilber Lourmat, Germany). The above dose of UVA reduced cell survival to about 70% (measured by the MTT test using 3-(4,5-dimethylthiazol-2-yl)-2,5-diphenyltetrazolium bromide) [77] and created pro-oxidative conditions [8]. In order to prevent heat stress, the surroundings of the plates were cooled with ice, and the cells to be irradiated were suspended in phosphate-buffered saline (PBS, 4 °C); the distance of the culture plates from the lamps was kept at 15 cm.

In order to study the effects of CBD (THC Pharm GmbH, Frankfurt, Germany) and/or CBG (Cayman Chemical Company, Ann Arbor, MI, USA) on keratinocytes (inspection and UVA irradiation), the following solutions of these phytocannabinoids were prepared: stock solution of CBD (32mM) was prepared by dissolving 1mg of CBD in 99.4 μL of ethanol (99.8%) and then diluting the prepared solution with ethanol to a concentration of 1.6 mM, which was added to the culture medium (3 μL/1ml of medium) to obtain a concentration of 5 μM, used into the keratinocyte culture medium; stock solution of CBG (32 mM) was prepared by dissolving 1 mg of CBG in 98.7 μL of ethanol (99.8%) and then diluting this solution with ethanol to a concentration of 0.33 mM, which was added to the culture medium (3 μL/1ml of medium) to obtain a concentration of 1 µM, used into the keratinocyte culture medium. To obtain a solution containing CBD and CBG, 10 µL of CBD stock solution and 2µL of CBG stock solution were mixed in 190µL of ethanol and further proceeded as above. Cells were cultured for 24 h in media containing CBD and/or CBG at concentrations of 5 µM and 1 µM, respectively, which were selected according to at least 85% viability checked by the MTT test [77]. Phytocannabinoid solutions were prepared in ethanol with a final concentration in the medium of 0.3%. After 24 h of incubation, control keratinocyte cells (control), UVA-irradiated keratinocytes (UVA) and phytocannabinoid-treated keratinocytes (including CBD; CBG; CBD+CBG group) and groups of keratinocytes irradiated with UVA and treated with phytocannabinoids (UVA+CBD; UVA+CBG; UVA+CBD+CBG) were washed 3 times with PBS (4 °C), collected by scraping and sonicated in cold PBS (4 °C) on ice. The samples were then centrifuged, and the resulting precipitate was used for further research. The total protein content of the cell lysate was measured by the Bradford test [78]. Experiments were carried out on eight research groups, with cells from the same group grown in 6 different plates/wells to obtain 6 replicates (n = 6).

### 4.2. Analysis of Keratinocyte Membrane Component Phospholipids

#### 4.2.1. Lipid Extraction and Quantification of Total Phospholipid Content

Total lipids were extracted from cell pellets with the use of the Bligh and Dyer method [79]. Quantification of the amounts of phospholipids in each extract was performed according to the Bartlett and Lewis method [80]. All experimental procedures for lipid extraction and phospholipid quantification were described in detail in a previously published paper [25].

#### 4.2.2. Phospholipid Profiling by Hydrophilic Interaction Liquid Chromatography Coupled with High-Resolution Tandem Mass Spectrometry

Phospholipids were separated by hydrophilic interaction liquid chromatography using a UPLC system (Agilent 1290; Agilent Technologies, Santa Clara, CA, USA) coupled with a QTOF mass spectrometer (Agilent 6540; Agilent Technologies, Santa Clara, CA, USA). Internal standards of PC (14:0/14:0), LPC (19:0), PE (14:0/14:0), PI (16:0/16:0) and PS (14:0/14:0) were used for the quantification and assessment of the ion variations. The mixture composed of solvent A (ACN/MeOH/water 50:25:25 (*v*/*v*/*v*) with 1 mM ammonium acetate) and solvent B (ACN/MeOH 60:40 (*v*/*v*) with 1 mM ammonium acetate) was used as the mobile phase. Gradient elution was applied starting with 0% A, increased linearly to 100% A within 20 min and held for 15 min, and then returned to 0% A in 10 min. Then, 25 μg of each phospholipid extract corresponding to a volume of 10 μL was mixed with 90 μL of the mobile phase (60% of A and 40% of B). A volume of 10 μL of the diluted sample was loaded into an Ascentis Si column (15 cm × 1 mm, 3 μm, Sigma-Aldrich) with a mobile-phase flow rate of 40 μL per min. The QTOF mass spectrometer was operated using the negative-ion mode (electrospray voltage, −3000 V) with a capillary temperature of 250 °C and a sheath gas flow of 13 L/min. The data-dependent acquisition mode (DDA) was used for data collecting in the range of *m*/*z* 100–1500 with a fixed collision energy of 35 eV. The LPE, PE, PI and PS species were analyzed as [M − H]^−^ ions, while LPC, PC and SM species were analyzed as [M + CH_3_COO]^−^ adducts. Data acquisition was carried out with the use of Mass Hunter data software (version B0.8.0, Agilent Technologies, Santa Clara, CA, USA). The relative content of each phospholipid ion species was achieved by normalizing the area of each peak to the peak area of the corresponding internal standard. The retention times and obtained MS/MS spectra were the basis of phospholipid identification.

#### 4.2.3. Ceramide Profiling by Reversed-Phase Chromatography Coupled with High-Resolution Tandem Mass Spectrometry RPLC-MS/MS Analysis of Ceramides

An Agilent UPLC-ESI-QTOF-MS system (Agilent 1290; Agilent 6540; Agilent Technologies, Santa Clara, CA, USA) was used to characterize the CER profiles. The mobile phase was composed of solvent A (water with 20 mM ammonium formate, pH 5) and solvent B (methanol). Initially, 70% B was held isocratically for 1 min, followed by a linear increase to 100% B within 75 min and a return to initial conditions in 5 min. The ceramides were separated on an RP C18 column (Acquity BEH Shield 2.1 mm × 100 mm; 1.7 μm; Waters, Milford, MA, USA) with a flow rate of 0.5 mL/min. The QTOF mass spectrometer was operated in positive-ion mode (electrospray voltage 3.5 kV) with a capillary temperature of 300 °C and a sheath gas flow rate of 8 L/min. The data were collected in DDA mode. Identification of ceramide species was based on the presence of the [M + H]+ molecular ion, retention time and characteristic fragmentation patterns observed in MS/MS spectra, which were previously described in detail [81].

#### 4.2.4. Determination of Sialic Acid Level

The modified Svennerholm’s resorcinol method was used to determine the total sialic acid content in keratinocyte membranes [82]. Color intensity was measured at 630 nm using a diode array spectrophotometer (Hewlett Packard, Palo Alto, CA, USA). The sialic acid concentration was read from the standard curve of the N-acetylneuraminic acid solution and recalculated as mg of protein.

### 4.3. Evaluation of Lipid Peroxidation

Lipid peroxidation was evaluated by measuring the level of malondialdehyde (MDA). This aldehyde was determined and quantified as the *O*-(2,3,4,5,6-pentafluoro-benzyl)-oxime-trimethylsilane (*O*-PFB-oxime-TMS) derivative by gas chromatography–tandem mass spectrometry using a modified method from Luo et al. [83]. In short, the cell lysates were incubated for 60 min at room temperature with *O*-(2,3,4,5,6-pentafluoro-benzyl) hydroxylamine hydrochloride (0.05 M in PIPES buffer, 200 µL), and benzaldehyde-D6 was used as an internal standard (ISTD). The GC-MS system included a GC-7000 quadrupole tandem mass spectrometer, 7890A (Agilent Technologies, Palo Alto, CA, USA), equipped with an HP-5 ms capillary column (30 m × 0.25 mm × 0.25 µm). Analyses were performed by monitoring *m*/*z* 204.0 and 178.0 ions for MDA-PFB-TMS and the *m*/*z* 307.0 ion for the ISTD derivative [40,84]. Any results obtained were recalculated as mg of protein.

### 4.4. Determination of Surface Charge Density

The electrophoretic mobility of keratinocyte cell membranes was determined using the Zetasizer Nano ZS apparatus (Malvern Instruments, Malvern, UK), as described previously [85]. The surface charge density was determined from electrophoretic mobility using the following formula:

$$\delta =\frac{\eta \cdot u}{d}$$

where u is the electrophoretic mobility, η is the viscosity of the solution, and d is the diffuse layer thickness. The diffuse layer thickness was determined from the following formula $d=\sqrt{\frac{\varepsilon \cdot \varepsilon_{0}\cdot R\cdot T}{2\cdot F^{2}\cdot I}}$, where ε·ε_0_ is the permeability of the electric medium, R is the gas constant, T is temperature, F = 96,487 (C∙mol^−1^) is the Faraday constant, and I is the ionic strength of 0.9% NaCl.

### 4.5. Measurements of Membrane Fluidity

Changes in membrane fluidity in the analyzed cells were studied using fluorescence staining by TMA-DPH (staining hydrophilic parts of the cell membrane) and DPH (anchored in the deeper, hydrophobic chains of cell membrane phospholipids). Fluorescence labels were dissolved in methanol (TMA-DPH) and THF (DPH). Labeled probes were well vortex-mixed and incubated for 20 min (t = 25 °C). Then, fluorescence anisotropy (r) was measured for both TMA-DPH (λ_exc._ = 340 nm, λ_em._ = 430 nm) and DPH (λ_exc._= 348 nm, λ_em._ = 426 nm). The values of fluorescence anisotropy were automatically calculated by the Perkin-Elmer (LS-55B) spectrofluorometer software (FL WINLAB version. 4.00.02) according to the Jablonski equation (Equation (1)) [86,87]:

(1) $$r=\frac{I_{VV}-GI_{VH}}{I_{VV}+2GI_{VH}}$$

where I_VH_ and I_VV_ are, respectively, the vertical values of the fluorescence intensity of the horizontal and vertical polarizations of the excitation light beam, while the G factor is the grating correction factor correcting the polarizing effects of the monochromator.

### 4.6. Analysis of Lipid Raft Formation Using Fluorescence Technique

To study lipid raft formation in the investigated cells, the d-4-ANEPPDHQ (dissolved in DMSO) fluorescent marker was used, which binds to the polar region of phospholipids. The label was added to cells suspended in 10 mM PBS (pH = 7.4) at a final concentration of 1 µM. The prepared samples were well vortex-mixed and incubated for 20 min at 25 °C. Lipid raft formation was analyzed according to Czajkowska-Szczykowska et al. [87].

### 4.7. Membrane Permeability

In order to assess changes in the permeability of the keratinocyte membrane under the influence of physical (UVA) and chemical (phytocannabinoids) factors, the efflux of lactate dehydrogenase (LDH) from the cells into the medium was determined [77]. LDH activity in the medium was determined based on changes in the NADH level in the presence of pyruvate, measured spectrophotometrically (340 nm using Multiskan GO Microplate Spectrophotometer, Thermo S. Scientific, Waltham, MA, USA). The amount of LDH released from the cells was calculated by comparing the activity in the medium with that of the whole-cell lysate.

### 4.8. Transmembrane Transporter Activity

The activity of membrane ABC-cassette transporters (human multidrug resistance 1 (MDR1), multidrug resistance protein (MRP1) and breast cancer resistance protein (BCRP)) was determined using the MDR test according to the manufacturer’s protocol (eFluxx-ID Multidrug resistance assay kits, Enzo LifeSciences, Exeter, UK). Keratinocytes with inhibitors of ABC transporters in DMSO and cells without inhibitors (PBS and DMSO contained in the buffer were approx. 1%) were incubated in dark 96-well plates for 5 min (37 °C). Then, green detection reagent was added, and after 30 min (37 °C) incubation, the fluorescence was measured (λex485 nm/λem535 nm) using the EnSpire 2300 Multilabel Reader (PerkinElmer, Waltham, MA, USA). The activities of MDR1, MRP and BCRP were normalized to the level of total protein, and the final results were expressed as the percentage of the activity of transporters compared to control cells.

### 4.9. Membrane Receptor Levels

An immunosorbent assay (ELISA) was used to measure protein expression in keratinocytes [13]. Keratinocyte lysates were incubated in an ELISA plate (Nunc Immuno MaxiSorp, Thermo Scientific, Waltham, MA, USA) for 3 h at 40 °C with blocking solution (skim milk—5% in carbonate binding buffer). Lysate supernatants were washed with PBS (with 0.1% Tween 20) and then incubated with primary antibodies at 40 °C for 24 h (CB1, CB2—host—mouse (Santa Cruz Biotechnology, Santa Cruz, CA, USA); PPARγ—host—rabbit (Invitrogen, Waltham, MA, USA)). Samples were washed with PBS (with 0.1% Tween 20) and then incubated with peroxidase blocking solution (3% H_2_O_2_) at room temperature for 30 min. After 1h incubation (1 h) with secondary goat anti-rabbit/mouse EnVision+ Dual Link/HRP solution (1:100) (Agilent Technologies, Santa Clara, CA, USA) at room temperature, antibodies were removed, and samples were incubated for 40 min with substrate solution (0.1 mg/mL TMB, 0.012% H_2_O_2_). Then, 2M H_2_SO_4_ was added to the samples to block the reaction. Absorbance (450 nm) was read after 10 min. Protein levels were determined from a calibration curve (CB1: Abcam, Cambridge, UK; CB2: Abnova, Taipei, Taiwan; PAPRγ: Sino Biological, Beijing, China).

### 4.10. Statistical Analysis

The data obtained in this study are expressed as median ± SD. Data were analyzed using the Kruskal–Wallis test with post hoc Dunn’s multiple comparison tests for multiple comparisons to identify significant differences between groups. *p* values < 0.05 were considered significant. Statistical analyses were performed using GraphPad Prism for Windows version 7.0.0 (GraphPad software, San Diego, CA, USA).

## 5. Conclusions

Since UVA radiation modifies the composition, structure and functionality of the lipid bilayer of keratinocyte membranes, the use of natural compounds, especially lipophilic compounds such as phytocannabinoids, is important for maintaining the proper condition of the skin and, consequently, for the proper functioning of the skin over the entire human body. Phytocannabinoids (CBD and CBG) have a protective effect on the structure and function of the cell membrane of keratinocytes exposed to UVA radiation. In addition, the reactions of the structural components of the lipid bilayer of the keratinocyte membrane, and thus changes in membrane fluidity, depend on the phytocannabinoids used. Based on the obtained results, it can also be suggested that the reduction in changes in the levels of phospholipid components of keratinocyte membranes may also be the result of reducing pro-oxidative conditions and lipid peroxidation by the phytocannabinoids used. The consequence of these structural changes is the reduction in functional changes in the permeability of cell membranes, especially under the influence of CBG and the CBG-CBD system. Phytocannabinoids have also been found to modify the activity of membrane transporters (ABC) in various ways, with CBG alone or in combination with CBD being particularly potent. Therefore, attention should be paid to the potentially protective effect of both phytocannabinoids on the membranes of keratinocytes irradiated with UVA radiation, while it should be emphasized that the action of CBG is multidirectional, in relation to both lipids and membrane proteins. This effect may be taken into account in the future for the design of new therapeutic preparations for application to the skin. However, before that, additional studies with a broader methodological aspect will be needed to confirm the potentially beneficial effects of phytocannabinoids, including in vivo studies.

## Data Availability

The data presented in this study are contained within the article.

## Abbreviations

2-AG	2-Arachidonoylglycerol
AEA	Anandamide
ANOVA	Analysis of variance
BCRP	Breast cancer resistance protein
CBD	Cannabidiol
CBG	Cannabigerol
CER[NDS]	Ceramides containing non-hydroxy fatty acids and dihydrosphingosine
CER[NS]	Ceramides containing non-hydroxy fatty acids and sphingosine
COX-2	Cyclooxygenase-2
CRAC	Calcium-release-activated calcium channel
DPH	1,6-Diphenyl-1,3,5-hexatriene
HDF	Human skin fibroblasts
IL	Interleukin
iNOS	Inducible nitric oxide synthase
Kv1.3	Potassium channel
LDH	Lactate dehydrogenase
MAPK	Mitogen-activated protein kinase
MDA	Malondialdehyde
MDR1	Multidrug resistance protein 1
MRP	Multidrug-resistance-associated proteins
MTT	3-(4,5-Dimethylthiazol-2-yl)-2,5-diphenyltetrazolium bromide
NHEK	Normal human epidermal keratinocytes
factors Nrf2	Nuclear factor erythroid 2-related factor 2
PC	Phosphatidylcholine
PI	Phosphatidylinositol
(PI3K)/AKT	Phosphatidylinositol 3-phosphatidyl kinase
PS	Phosphatidylserine
ROS	Reactive oxygen species
SM	Sphingomyelin
TMA-DPH	N,N,N-Trimethyl-4-(6-phenyl-1,3,5-hexatrien-1-yl)phenylammonium p-Toluenesulfonate
TNF-α	Tumor necrosis factor alpha
TNFR	Tumor necrosis factor receptor
UVA	Ultraviolet A
